# Structural, Electronic, and Optical Properties of CsPb(Br_1−x_Cl_x_)_3_ Perovskite: First-Principles Study with PBE–GGA and mBJ–GGA Methods

**DOI:** 10.3390/ma13214944

**Published:** 2020-11-03

**Authors:** Hamid M. Ghaithan, Zeyad. A. Alahmed, Saif M. H. Qaid, Abdullah S. Aldwayyan

**Affiliations:** 1 Physics and Astronomy Department, College of Science, King Saud University, P.O. Box 2455, Riyadh 11451, Saudi Arabia; sqaid@ksu.edu.sa; 2 King Abdullah Institute for Nanotechnology, King Saud University, P.O. Box 2454, Riyadh 11451, Saudi Arabia; 3 K.A.CARE Energy Research and Innovation Center at Riyadh, P.O. Box 2022, Riyadh 11454, Saudi Arabia

**Keywords:** CsPb(Br_1−x_Cl_x_)_3_ perovskite, PBE–GGA and mBJ–GGA, structural properties, electronic properties, optical properties

## Abstract

The effect of halide composition on the structural, electronic, and optical properties of CsPb(Br_1−x_Cl_x_)_3_ perovskite was investigated in this study. When the chloride (Cl) content of x was increased, the unit cell volume decreased with a linear function. Theoretical X-ray diffraction analyses showed that the peak (at 2θ = 30.4°) shifts to a larger angle (at 2θ = 31.9°) when the average fraction of the incorporated Cl increased. The energy bandgap (E_g_) was observed to increase with the increase in Cl concentration. For x = 0.00, 0.25, 0.33, 0.50, 0.66, 0.75, and 1.00, the E_g_ values calculated using the Perdew–Burke–Ernzerhof potential were between 1.53 and 1.93 eV, while those calculated using the modified Becke−Johnson generalized gradient approximation (mBJ–GGA) potential were between 2.23 and 2.90 eV. The E_g_ calculated using the mBJ–GGA method best matched the experimental values reported. The effective masses decreased with a concentration increase of Cl to 0.33 and then increased with a further increase in the concentration of Cl. Calculated photoabsorption coefficients show a blue shift of absorption at higher Cl content. The calculations indicate that CsPb(Br_1−x_Cl_x_)_3_ perovskite could be used in optical and optoelectronic devices by partly replacing bromide with chloride.

## 1. Introduction

Over the last decade, organic and inorganic perovskites have gained considerable attention in the field of optoelectronics, and more recently in solar cells [1,2,3,4,5,6,7,8] and light-emitting devices [9,10,11,12,13], thanks to the reduced costs [14], high quantum efficiency of photoluminescence [15], and extensively tunable emission wavelengths of these materials [16,17,18]. Recently, inorganic mixed-halide CsPb(Br_1−x_Cl_x_)_3_ compositions were used for creating various nanophotonic components because they exhibit electroluminescence in the green [12,19] to blue [20] optical ranges. CsPbBr_3_ exhibits orthorhombic symmetry at temperatures below 88 °C. When the temperature increases, structural distortion occurs and the structure of CsPbBr_3_ is converted to tetragonal (88 °C < T < 130 °C), and subsequently to cubic at higher temperatures (T > 130 °C) [17,18,21,22,23,24,25,26,27,28,29,30,31,32,33,34,35,36,37,38]. In comparison, at temperatures below 42 °C, CsPbCl_3_ exhibits orthorhombic symmetry. When temperature increases, structural distortion occurs and the CsPbCl_3_ structure is converted to tetragonal (42 °C < T < 47 °C), and subsequently to cubic at higher temperatures (T > 47 °C) [18,39]. The energy band gap (E_g_) can be adjusted by adding appropriate materials to the perovskite, which can be designed using theoretical simulations based on density functional theory (DFT) [40]. Recent studies on CsPb(Br_1−x_Cl_x_)_3_ perovskite thin films, fabricated by sequential deposition technique, revealed an orthorhombic lattice in the case of x = 0.1 and 0.2, whereas for x = 0.4 and 0.6, a cubic phase was observed [41]. The electronic structure of CsPb(Br_1−x_Cl_x_)_3_ perovskites was studied theoretically and experimentally by Tatiana G. Liashenko et al. [18]. Cl ions, which are the substitute for Br ions in the perovskite crystal lattice at room temperature, do not change its orthorhombic symmetry [18]. Generally, theoretical investigations of electronic and optical properties of organic-inorganic perovskites are often performed by first-principles calculations with the local density approximation (LDA) [42] and Perdew–Burke–Ernzerhof generalized gradient approximation (PBE–GGA) [43,44] using DFT because of their relatively cheap computational cost and reasonable accuracy [45]. The LDA and PBE–GGA potentials failed to calculate the accurate E_g_ and optical properties because the obtained E_g_ values were much smaller than the experiment values [43,44,46,47,48] and other possible errors [45]. In addition, the theoretical lattice parameters calculated using PBE–GGA overestimated the experimental lattice constants [45]. LDA potential usually underestimated the lattice constants, which resulted in the underestimation of E_g_ [45]. To overcome these significant problems of LDA and PBE–GGA potentials, the most accurate potential modified Becke−Johnson GGA (mBJ–GGA) potential was used, which is much more accurate than all other semi-local potentials for strongly correlated systems [49,50]. mBJ–GGA potential can be used for the calculation of E_g_ with excellent agreement with experimental values thanks to its additional dependence on kinetic energy density [49,50].

In this study, the effects of substituting Cl with Br on the structural, electronic, and optical properties of mixed Br–Cl supercell 1 × 1 × 4 CsPb(Br_1−x_Cl_x_)_3_ (x = 0.00, 0.25, 0.33, 0.50, 0.66, 0.75, and 1.00) are investigated using PBE–GGA and mBJ–GGA potentials. The calculated values were compared to the previous experimental [51,52,53,54,55,56] and theoretical [27,33,57,58,59,60,61,62,63,64,65,66,67,68] results to verify the validity of the DFT calculation. The effect of spin-orbital coupling (SOC) [57,58,59,60] was included in the calculation because of the heavy lead (Pb) element. By increasing the Cl content x from 0.00 to 1.00, the lattice constants and E_g_ were calculated. In addition, for these mixed-halide perovskites, the effective masses of charge carriers, the binding energy of the exciton, the absorption coefficients, the optical conductivity, the dielectric constants, and the reflectivity were calculated in detail.

## 2. Computational Method

The full-potential linearized augmented plane wave method [61,62] based on DFT [63], as implemented in the WIEN2k code [64], has been used in the calculation. The structural properties for CsPb(Br_1–x_Cl_x_)_3_ (x = 0.00, 0.25, 0.33, 0.50, 0.66, 0.75, and 1.00) were performed using Wu and Cohen (GGA–WC) potential [65]. For the electronic and optical properties, mBJ–GGA [66] and PBE–GGA potentials were used [67]. The mBJ–GGA potential with the SOC effect was included in our DFT calculation because of the heavy Pb element.

The R_MT_* k_max_ value was set at 9.0 (R_MT_ is the smallest muffin-tin radius in the unit cell and k_max_ is the maximum value of the reciprocal lattice vectors). The R_MT_ values were set at 2.5 a.u for (Cs, Pb, and Br) and 2.41 a.u for Cl in such a way that the muffin-tin spheres do not overlap. To ensure the accuracy of our calculations, we considered G_max_ = 12 and l_max_ = 10. The irreducible Brillouin zone (IBZ) was produced using 500 k-points (12 × 12 × 3 mesh grids) and the self-consistent convergence of total energy was set at 10^−4^ Ry.

## 3. Results

### 3.1. Structural Properties

CsPbBr_3_ and CsPbCl_3_ have cubic structures with space group Pm$\bar{3}$m (no. 221); the unit cell contains one formula unit. To simulate CsPb(Br_1−x_Cl_x_)_3_, a tetragonal 1 × 1 × 4 supercell with 20 atoms was used. For x = 0.00, 0.25, 0.33, 0.50, 0.66, 0.75, and 1.00, a supercell with 0, 3, 4, 6, 8, 9, and 12 atoms of bromide was substituted with chloride atoms, respectively. See the Supplementary Materials, Tables S1–S7, for more details.

**Figure 1:**
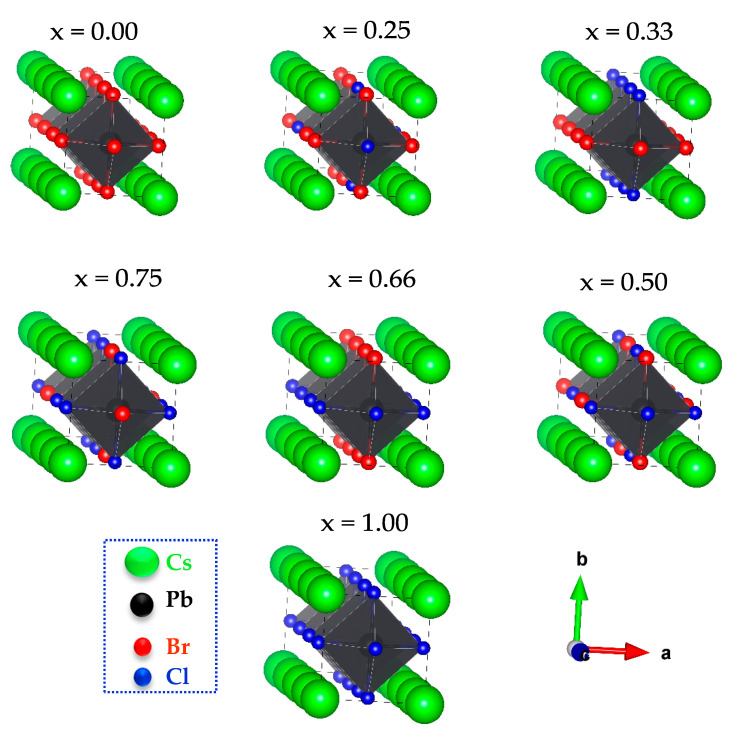
Atomic structures of CsPb(Br_1−x_Cl_x_)_3_, with x = 0.00, 0.25, 0.33, 0.50, 0.66, 0.75, and 1.00 for different Cl content (x).

Figure 1 shows the crystal structure of 1 × 1 × 4 supercell CsPb(Br_1−x_Cl_x_)_3_ formed by cubic CsPbBr_3_ and CsPbCl_3_.

**Figure 2:**
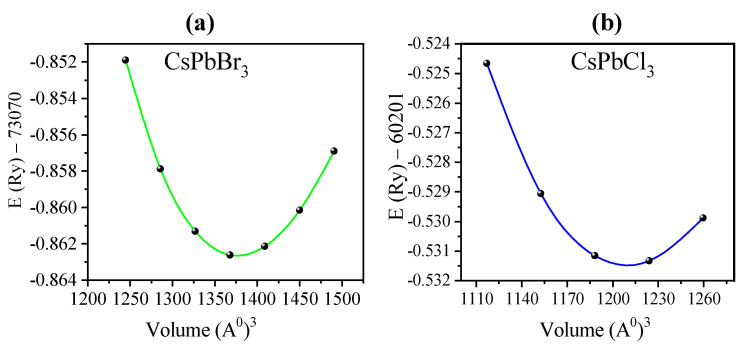
Calculated total energy versus volume of (**a**) CsPbBr_3_ and (**b**) CsPbCl_3_ via (Wu and Cohen generalized gradient approximation (WC–GGA)) potential.

The WC–GGA potential was determined by evaluating the ground state properties. These properties include the lattice constant *a*, bulk modulus *B*, and its pressure derivative *B′.* Structural optimization was performed by minimizing total energy with respect to cell volume, and the results of total energy versus unit-cell volume were fitted with Murnaghan’s state-of-the-art equation [68]. The total energy versus volume graph is shown in Figure 2. The results of *a*, *B*, and *B′* are shown in Table 1 with the corresponding theoretical and experimental data available in the literature. As shown in Table 1, the lattice constants of the CsPbBr_3_ and CsPbCl_3_ structures are in good agreement with recent theoretical and experimental results, thereby proving that our computational parameters are valid.

**Figure 3:**
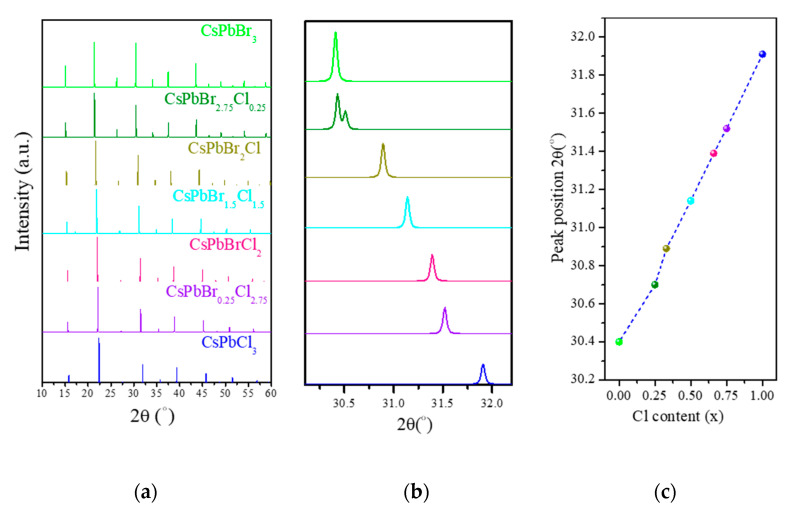
(**a**) Theoretical X-ray diffraction (XRD) patterns of CsPb(Br_1−x_Cl_x_)_3_ obtained using visualization for electronic and structural analysis (VESTA) software, (**b**) XRD patterns (2θ = 30°–32.1°), and (**c**) the peak position versus Cl content (x).

**Figure 4:**
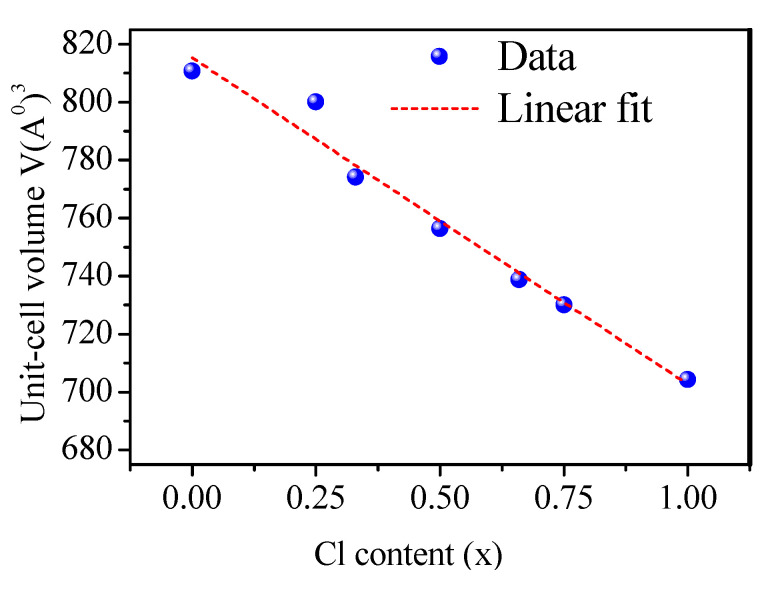
Unit-cell volume versus Cl content (x).

Moreover, excellent agreement was observed between our obtained value of the lattice parameter for CsPbBr_3_ (5.8859 Å) and its experimental value of 5.85 (Å) obtained in [69]. Moreover, the value of the lattice parameter for CsPbCl_3_ was 5.6379 Å, which was in excellent agreement with the experimental value of 5.605 Å obtained in [70]. Theoretical X-ray diffraction (XRD) patterns were obtained using the visualization for electronic and structural analysis (VESTA 3, Ibaraki, Japan) [71] (see Figure 3). The diffraction peaks of CsPbBr_3_ moved toward CsPbCl_3_ when x changed from 0.00 to 1.00. As shown in Table 1, when the Cl content x increases from 0.00 to 1.00, the volume of the unit-cell decreases in proportion x with the function of V(x) = 815.29916 – 112.58513x (Å)^3^, as shown in Figure 4.

### 3.2. Electronic Properties

#### 3.2.1. Electronic Band Structure

**Figure 5:**
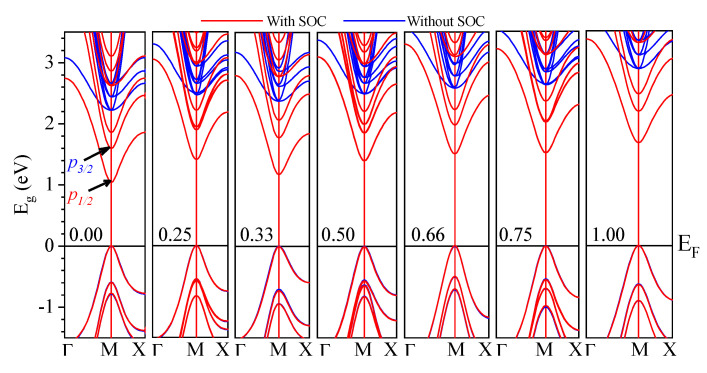
Calculated band structures of CsPb(Br_1–x_Cl_x_)_3_ using the modified Becke−Johnson (mBJ)-GGA potential without/with spin-orbital coupling (SOC).

**Figure 6:**
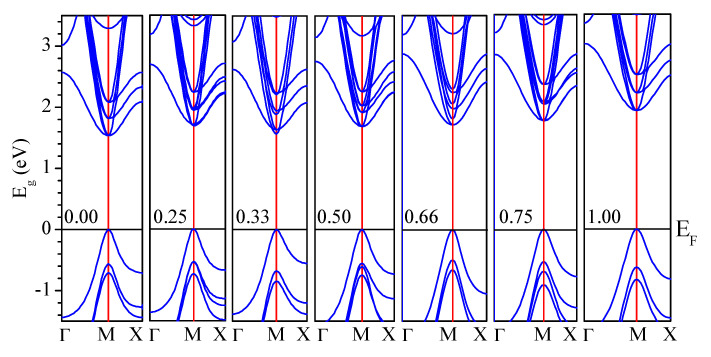
Calculated band structures of CsPb(Br_1−x_Cl_x_)_3_ using the Perdew–Burke–Ernzerhof (PBE)-GGA potential.

First, the electronic structures for CsPb(Br_1−x_Cl_x_)_3_ were calculated by PBE–GGA and mBJ–GGA potentials without/with SOC. Figure 5 shows the calculated band structures of CsPb(Br_1−x_Cl_x_)_3_ using the mBJ–GGA potentials without/with SOC. In contrast, Figure 6 shows those using the potential of PBE–GGA without SOC. The band structures have a direct transition character at M, which can improve the photoabsorption coefficient and accelerate the rate of radiative recombination [84]. The calculated E_g_ for CsPbBr_3_, CsPbBr_2.75_Cl_0.25_, CsPbBr_2_Cl, CsPbBr_1.5_Cl_1.5_, CsPbBrCl_2_, CsPbBr_0.25_Cl_2.75_, and CsPbCl_3_ based on the mBJ–GGA potential are 2.23, 2.46, 2.40, 2.51, 2.59, 2.64, and 2.90 eV, respectively, whereas the E_g_ values obtained using the PBE–GGA potential are 1.53, 1.68, 1.56, 1.69, 1.71, 1.77, and 1.93 eV, respectively, as shown in Table 2. The E_g_ calculated using mBJ–GGA were the closest to the experimental values [51,52,53,54,55].

**Figure 7:**
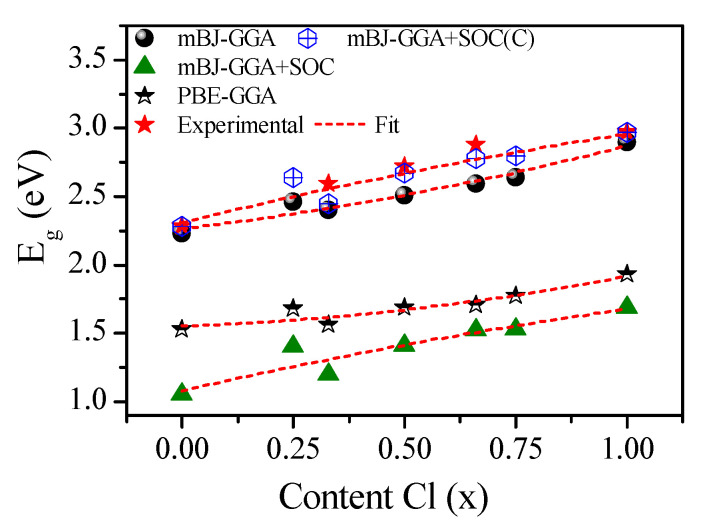
Band gaps of CsPb(Br_1−x_Cl_x_)_3_ using the PBE–GGA and mBJ–GGA potentials with/without SOC. By applying the band gap correction, we get the mixed ratio band gaps of inorganic mixed halide perovskite compared with the experimental results.

By including the effect of SOC, the calculated E_g_ values are smaller than the experimental by approximately 1.23 and 1.28 eV for pure CsPbBr_3_ and CsPbCl_3_, respectively, and result in more reasonable band dispersions [85,86]. The SOC causes the conduction band (CB) to decrease by splitting it into a twofold degenerated state (p_1/2_) corresponding to light electrons and a fourfold degenerate state (p_3/2_) corresponding to heavy electrons at this point [57,87,88]. In contrast, the valance band (VB) showed no significant change in this area [57,87,88]. The correction was thus applied to the E_g_ with the following equation [78,84,89]:

(1) $$\Delta E_{g}\;\left(A_{1-x}B_{x}\right)=\left(1-x\right)\Delta E_{g}\left(A\right)+x\Delta E_{g}\left(B\right)$$

where $\Delta E_{g}\;\left(A_{1-x}B_{x}\right)$, $\Delta E_{g}\left(A\right)$, and $\Delta E_{g}\left(B\right)$ are the E_g_ corrections for the CsPb(Br_1−x_Cl_x_)_3_, CsPbBr_3_, and CsPbCl_3_ compounds, respectively. Figure 7 shows the calculated E_g_ using PBE–GGA, mBJ–GGA, mBJ–GGA + SOC, and corrected mBJ–GGA + SOC(C). The calculated E_g_ by mBJ–GGA and mBJ–GGA + SOC(C) are in good agreement with the experimental values [53,55]. The small differences between the theoretical and experimental values are mainly attributed to the changed size for different mixed-halide [84], as depicted in the XRD patterns and the small 1 × 1 × 4 supercell models.

The optical bowing parameter (b) was calculated for determining the relationship between the E_g_ and the Cl composition x [78,90,91] using the following equation:

(2) $$\Delta E_{g}\left(x\right)=\mathrm{bx}\left(x-1\right)={\;E}_{g}\left(x\right)-[\left(1-x\right)E_{g}\left(A\right)+{\mathrm{xE}}_{g}\left(B\right)$$

where b is the bowing parameter; E_g_(A) and E_g_(B) are the band gaps of pure A and B, respectively; and E_g_(x) is the bandgap of A, B mixed-halide perovskites with the composition x. The dependence of the obtained E_g_ on the concentration of Cl (x) was given by fitting the nonlinear variation with the quadratic function as follows:

(3) $$E_{g}^{\left(\mathrm{PBE}-\mathrm{GGA}\right)}\left(x\right)=1.55235+0.11317\;x+0.25154{\;x}^{2}$$

(4) $$E_{g}^{\left(\mathrm{mBJ}-\mathrm{GGA}\right)}\left(x\right)=2.26601+0.37552\;x+0.23037{\;x}^{2}$$

(5) $$E_{g}^{\left(\begin{array}{c} mBJ-GGA\\ SOC \end{array}\right)}\left(x\right)=1.08065+0.73866\;x-0.14156{\;x}^{2}$$

(6) $$E_{g}^{\left(\begin{array}{c} mBJ-GGA\\ SOC\left(C\right) \end{array}\right)}\left(x\right)=2.31016+0.79639\;x-0.14902{\;x}^{2}$$

These results indicate the bowing parameters b= 0.25154, 0.23037, −0.14156, and −0.14902 eV for the E_g_ obtained using PBE–GGA, mBJ–GGA, mBJ–GGA + SOC, and mBJ–GGA + SOC(C), respectively.

**Figure 8:**
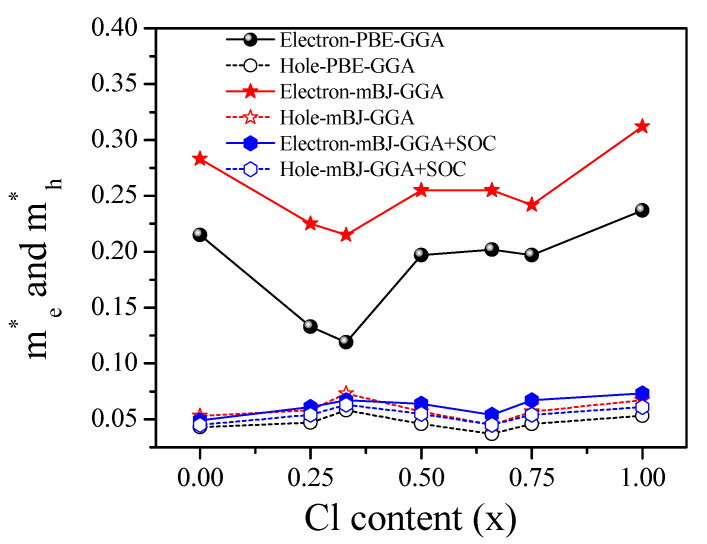
Effect of Cl concentration on the electron and hole effective masses for CsPb(Br_1−x_Cl_x_)_3_ perovskites.

The influences of the dispersive nature of the conduction band (CB) and valence band (VB) on the effective masses (${m_{e}}^{*}$ and ${m_{h}}^{*}$) are shown in Figure 8. The effective masses are related to carrier mobility, which is an essential criterion for the excellent power efficiency of photovoltaic materials [85]. ${m_{e}}^{*}$ and ${m_{h}}^{*}$ at the band edges are related to the band dispersions. As a result, the effective masses at the CB minimum (CBM) and VB maximum (VBM) were approximated by a parabola [85,97,98,99]. By fitting the VB and CB edges, the effective mass (m^∗^) was evaluated numerically using the following equations:

(7) $${\left(m^{*}\right)}_{ij}=\;\hbar^{2}{\left[\frac{\partial^{2}\varepsilon_{n}\left(\;\vec{k}\right)}{\partial k_{i}k_{j}}\right]}^{-1}\;i,\;j\;=\;x,\;y,\;z$$

where $m^{*}$ is the effective mass of the charge carrier, *i* and *j* are the reciprocal components, $\varepsilon_{n}\left(\vec{k}\right)$ is the energy dispersion function of the *n*^th^ band, $\vec{k}$ represents the wave vector, and ℏ represents the reduced Planck constant.

The mBJ–GGA calculation without SOC results in an accurate E_g_ value; however, the previous studies stated that the introduction of SOC increases band dispersion and results in more accurate effective masses with respect to DFT calculation without SOC [23,78,79,86,92,96,100,101,102]. Therefore, we employ mBJ–GGA + SOC to evaluate the effective charge masses. The values of m_e_* and m_h_* decreased significantly with the increase in Cl concentration up to 0.33 owing to the decrease of parabolic nature of the band structure [103]. The increased parabolic nature caused a drastic increase of the effective mass of carriers for high concentration of Cl [103]. The calculated effective charge masses around the M point of the Brillouin zone obtained by evaluating the second derivatives are shown in Table S8 (Supplementary Materials). The reduced masses $\mu_{r}$ were calculated using the following equation:

(8) $$\mu_{r}=\;\frac{{m_{e}}^{*}{m_{h}}^{*}}{{m_{e}}^{*}+{m_{h}}^{*}}$$

The effective Bohr diameter of a Wannier–Mott exciton (*a*_0_) can be defined [99] using the following equation:

(9) $$a_{0}=\;\frac{2\hbar^{2}\varepsilon \left(\infty \right)}{\mu_{r}e^{2}}$$

where ε(∞) is the dielectric constant in the limit of infinite wavelength, and the exciton binding energy (E_b_) is given by the following:

(10) $$E_{b}=\frac{2\hbar^{2}\varepsilon \left(\infty \right)}{\mu_{r}{a_{0}}^{2}}$$

For calculating E_b_, we need to know the dielectric constant of the material *ε*(*∞*) and the reduced masses (*μ*_r_), which can be obtained by DFT calculation. The estimated *a*_0_ and E_b_ values were between 5.6 and 8.9 nm and between 41 and 72 meV, respectively, which were in good agreement with other theoretical [16,75,100,104,105] and experimental [106,107] values. A weaker E_b_ indicates that the charge carriers behave more like free charge carriers [99].

The dependence of the obtained a_0_ and E_b_ values on the concentration of Cl (x) was determined by fitting the nonlinear variation as Cl concentration x with the linear and quadratic functions as follows:

(11) $$a_{0}^{\mathrm{PBE}-\mathrm{GGA}}\;\left(x\right)=13.6268-4.12266\;x$$

(12) $$a_{0}^{\mathrm{mBJ}-\mathrm{GGA}}\;\left(x\right)=8.16334-1.5024\;x$$

(13) $$E_{b}^{\left(\mathrm{PBE}-\mathrm{GGA}\right)}\left(x\right)=22.29863-0.92682\;x+17.41855{\;x}^{2}$$

(14) $$E_{b}^{\left(\mathrm{mBJ}-\mathrm{GGA}\right)}\left(x\right)=44.29984-24.98693\;x+49.22733{\;x}^{2}$$

**Figure 9:**
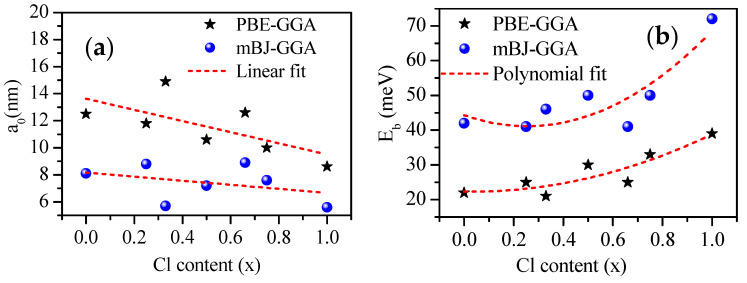
(**a**) Bohr diameter a_0_ (nm) and (**b**) exciton binding energy E_b_ (meV) with respect to Cl content (x).

These results indicate the Bohr diameter bowing parameters of b= −4.12266 and−1.5024 nm obtained using PBE–GGA and mBJ–GGA, respectively. These results show that a_0_ decreased with the increase in Cl concentration, as shown in Figure 9a. Furthermore, the bowing parameters b=17.41855 and 49.22733 meV of $E_{b}$ using PBE–GGA and mBJ–GGA indicated the decrease in $E_{b}$ with the increase in Cl concentration (x), as shown in Figure 9b.

#### 3.2.2. Density of States (DOS)

**Figure 10:**
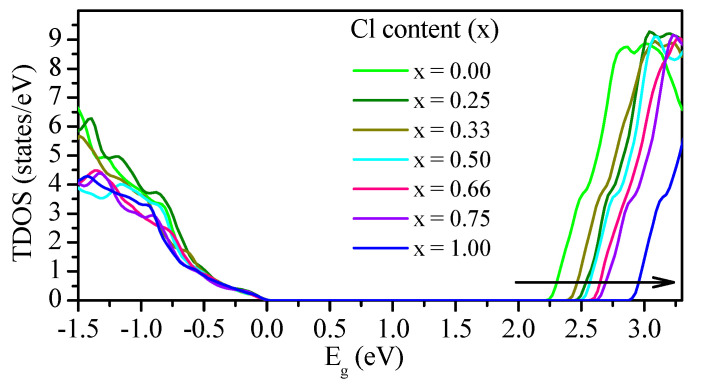
Total density of states (TDOS) of CsPb(Br_1−x_Cl_x_)_3_ calculated using the mBJ–GGA potential.

**Figure 11:**
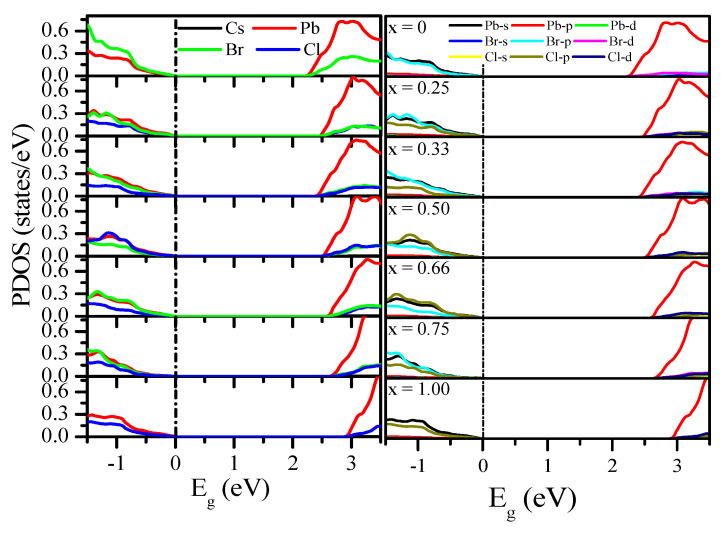
Calculated partial DOS (PDOS) of CsPb(Br_1−x_Cl_x_)_3_ calculated using the mBJ–GGA potential without SOC.

The total DOS (TDOS) was calculated using the mBJ–GGA potential, as shown in Figure 10. However, as the concentration (x) increased from 0.00 to 1.00, the DOS edges changed. The partial DOS (PDOS) shown in Figure 11 are based on the mBJ–GGA potential, because we are interested in the valence band (VB) and conduction band (CB) components. Previous studies have shown that inorganic cation Cs^+^ does not contribute to VB maximum (VBM) and CB minimum (CBM), and only maintains overall load neutrality and structural stability [23,26,37,72,75,78,79,82,85,92,93,100,101,108,109]. Therefore, we observed only the states of Pb and halogen elements (Cl and Br), as shown in Figure 11. The VBM originates mainly from the p orbitals of Br and Cl, and a small number of contributions from s orbitals of Pb can also be observed. The CBM originated from the p states of Pb and halogen elements (Cl and Br). The CB structure is relatively similar for all of the compounds, and the CBM for each compound comprises mainly p orbitals of Pb and halogen elements (Cl and Br). The uppermost VB is steep, while the lowermost CB in PDOS is relatively flat.

**Figure 12:**
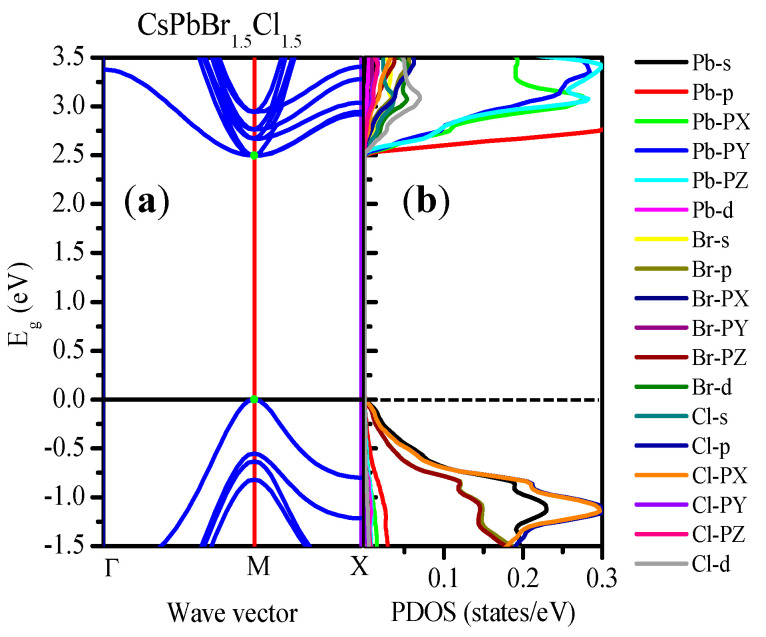
(**a**) Band structures and (**b**) PDOS of CsPbBr_1.5_Cl_1.5_ obtained using the mBJ–GGA potential.

For a detailed view of the band structure of CsPbBr_1.5_Cl_1.5_, PDOS was plotted on the band structure using the mBJ–GGA potential (Figure 12a). The PDOS (Figure 12b) indicated that the effects of the Cs atoms did not follow any specific rules, whereas it shows that the E_g_ trends are the result of the effects of Pb and Br [93]. Similar band structures of CsPbBr_3_ and CsPbCl_3_ with PDOS are shown in Figure S1 (Supplementary Materials).

**Figure 13:**
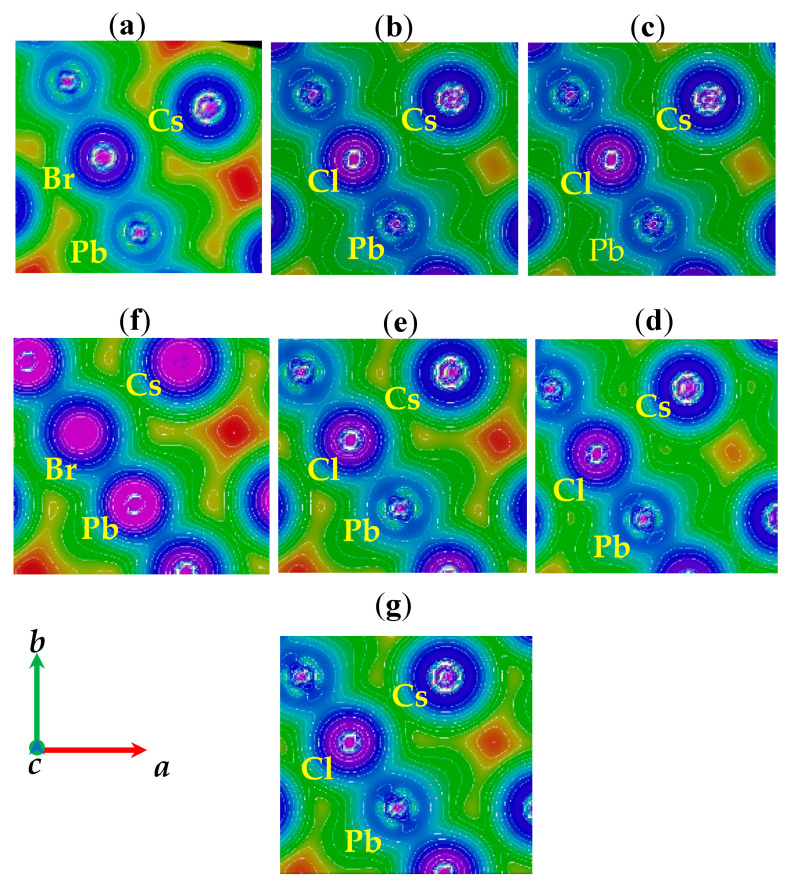
Calculated electron density in the (001) plane of CsPb(Br_1−x_Cl_x_)_3_. (**a**) x = 0.00, (**b**) x = 0.25, (**c**) x = 0.33, (**d**) x = 0.50, (**e**) x = 0.66, (**f**) x = 0.75, and (**g**) x = 1.00 using the mBJ–GGA potential.

To support this observation, the total charge density distributions are calculated and presented in the (001) plane, as shown in Figure 13a–g, with the structures adjacent to each concentration. The nature of bonding among the atoms could be analyzed using the map of electronic charge density distribution [72,109]. According to the Pauling scale, the electro-negativity of Cs, Pb, Br, and Cl is 0.79, 2.33, 2.96, and 3.16, respectively. For the description of the bonding character, the difference of the electro-negativity (X_A_-X_B_) is crucial [110], where X_A_ and X_B_ are the electro-negativities of the A and B atoms, respectively. The percentage of the ionic character (IC) of the bonding can be obtained from the following equation [111]:

(15) $$\%\;\mathrm{IC}=\left[1-e^{-\left(0.25\right){\left(X_{A}-X_{B}\right)}^{2}}\right]*100$$

Using this equation, the obtained % IC of Cs–Br, Cs–Cl, Pb–Br, and Pb–Cl was 69.85, 75.44, 10.02, and 15.82, which indicated that the bond between Cs–Cl/Br is mostly ionic and partially covalent. In contrast, the Pb–Cl/Br bond is mostly covalent and partially ionic. Strong covalent bonds between Pb-halides have also been predicted by previous reports [72,79,110,112].

### 3.3. Optical Properties

The study of the optical properties of the CsPb(Br_1−x_Cl_x_)_3_ perovskite is essential because of its potential for use in photonic and optoelectronic applications. Calculations of dielectric functions with both real ε_1_(ω) and imaginary ε_2_(ω) parts, refractive index n(ω), extinction coefficient k(ω), absorption coefficient α(ω), optical conductivity 𝜎(ω), and reflectivity R(ω) were explored by mBJ–GGA potential. These optical parameters can be attracted by the knowledge of the complex dielectric function ε(ω) = ε_1_ (ω) + iε_2_ (ω). The imaginary part of the dielectric function ε_2_ (ω), according to the perturbation theory, is given by the following equation [113,114]:

(16) $$\varepsilon_{2}\left(\omega \right)=\left(\frac{h^{2}e^{2}}{{\pi w}^{2}m^{2}}\right)\sum_{i,j}\int d^{3}k\langle i_{k}{|p}^{\alpha }{|j}_{k}\rangle \langle j_{k}{|p}^{\beta }{|i}_{k}\rangle x\delta \left(\varepsilon_{i_{k}}-\varepsilon_{j_{k}}-\omega \right)$$

where p is the moment matrix element between the band α and β states within the crystal momentum k. i_k_ and j_k_ are the crystal wave functions corresponding to the conduction and valence bands with the crystal wave vector k, respectively. The real part $\varepsilon_{1}\left(\omega \right)$ of the dielectric function can be expressed as follows [114]:

(17) $$\varepsilon_{1}\left(\omega \right)=1+\frac{2}{\pi }p\int_{0}^{\infty }\frac{\omega^{\prime }\varepsilon_{2}\left(\omega^{\prime }\right)}{{\left(\omega^{\prime }\right)}^{2}-{\left(\omega \right)}^{2}}d\omega$$

where p is the value of the principal of the integral.

The absorption coefficient, optical conductivity, refractive index, extinction coefficient, and reflectance denoted by α (ω), σ (ω), n (ω), k (ω), and R (ω), respectively, are directly related to the $\varepsilon_{1}\;\left(\omega \right)$ and $\varepsilon_{2}\;\left(\omega \right)\;$[113,114,115,116].

**Figure 14:**
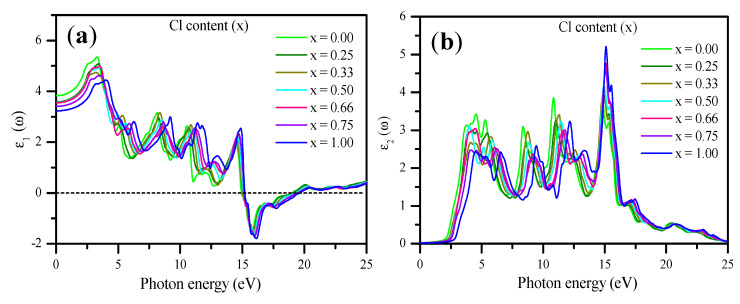
Calculated (**a**) real dielectric function ε_1_ (ω) and (**b**) imaginary dielectric function ε_2_ (ω) of CsPb(Br_1−x_Cl_x_)_3_ with respect to Cl content (x) using the mBJ–GGA potential.

The calculated $\varepsilon_{1}\;\left(\omega \right)$ and $\varepsilon_{2}\;\left(\omega \right)$ are shown in Figure 14a,b. As shown in Figure 14a, the static dielectric constant $\varepsilon_{1}\left(0\right)$ is given by the low energy limit of $\varepsilon_{1}\left(\omega \right)$. The peaks of $\varepsilon_{1}\left(\omega \right)$ shifted to higher energy as x increased from 0.00 to 1.00. The results obtained using mBJ–GGA for $\varepsilon_{1}\left(0\right)$ at various Cl concentrations (x) are presented in Table 3 and shown in Figure 17. $\varepsilon_{1}\left(0\right)$ decreased with an increase in the concentration of Cl, consistent with an increase in E_g_. The results obey the following equation:

(18) $$\varepsilon_{1}\left(x\right)=\;3.77052-0.4113\;x-0.09431\;x^{2}$$

For CsPbBr_3_, $\varepsilon_{1}\left(0\right)$ was 3.82, which agrees well with the result obtained in the previous studies [23,72,105]. Figure 14b shows the behavior of ε_2_ (ω) for all Cl concentrations. For x = 0.00, 0.25, 0.33, 0.50, 0.66, 0.75, and 1.00, the critical points in $\varepsilon_{2}\left(\omega \right)$ occurred at approximately 2.14, 2.25, 2.28, 2.34, 2.45, 2.55, and 2.84 eV, respectively, which were closely related to the direct E_g_ values of 2.26, 2.47, 2.39, 2.51, 2.58, 2.64, and 2.90 eV, respectively.

**Figure 15:**
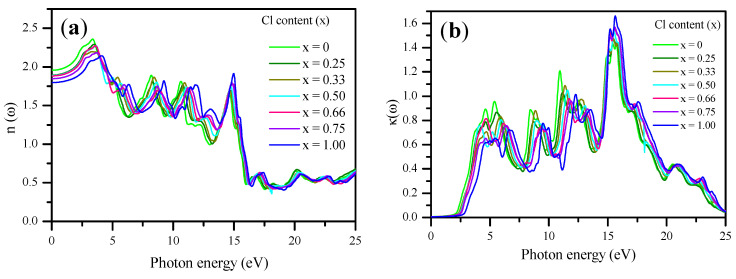
Calculated (**a**) refraction indices n (ω) and (**b**) extinction coefficients k (ω) of CsPb(Br_1−x_Cl_x_)_3_ with respect to Cl content (x) using the mBJ–GGA potential.

The refractive index n (ω) and extinction coefficients k (ω) were calculated using the mBJ–GGA potential, as shown in Figure 15a,b. The spectrum of n (ω) closely resembles the spectrum of $\varepsilon_{1}\left(\omega \right)\;$[117]. For CsPbBr_3_, the calculated n (0) value was 1.96, which agrees well with the previous theoretical and experimental values [56,72]. For CsPbCl_3_, n(0) was 1.798, which agrees well with the previous value [72,81]. The calculated n (0) versus the Cl concentration (x) is expressed as follows:

(19) $$n\left(x\right)=\;1.94752-0.13036\;x-0.00939\;x^{2}$$

Figure 15b shows that k(ω) depends on the concentration of Cl similar to that of ε_2_ (ω). The peak value of k (ω) shifted to lower energies as Cl concentration increased from 0.00 to 1.00.

**Figure 16:**
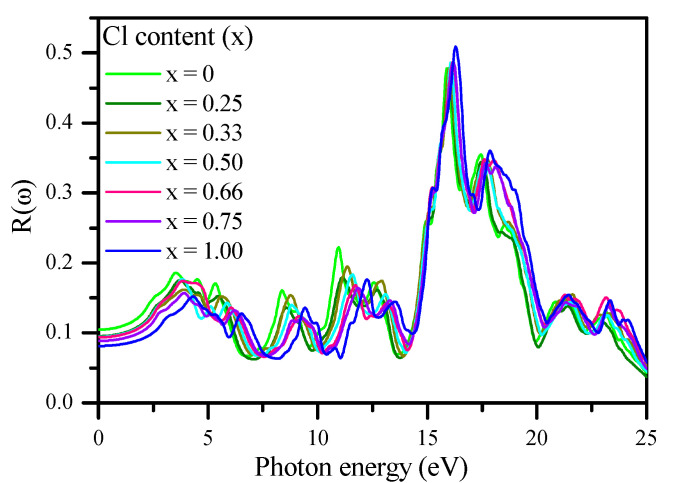
Calculated reflectivity spectra R (ω) of CsPb (Br_1−x_Cl_x_)_3_ using the mBJ–GGA potential.

**Figure 17:**
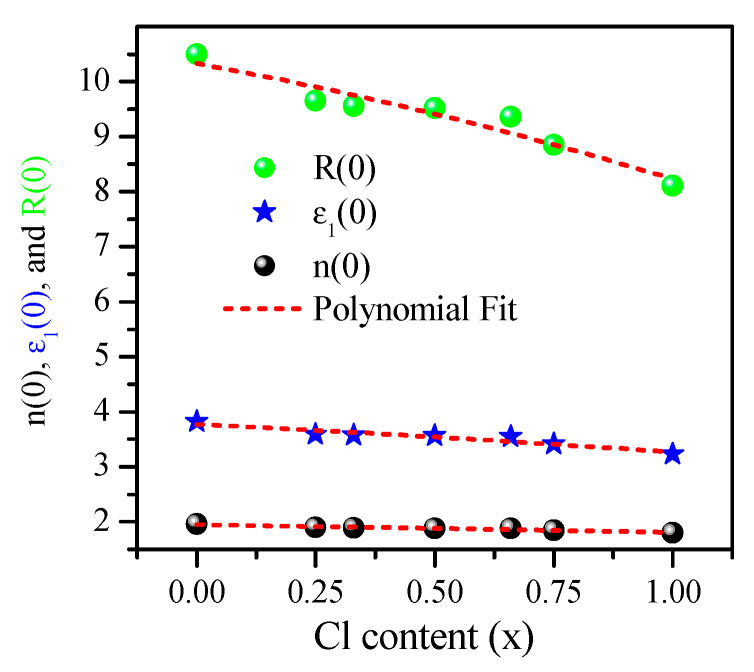
Static refractive index, real dielectric function, and reflectivity at zero frequency versus Cl content (x).

The initial reflectivity R(ω) values were around 10.50% and 8.11% at zero frequency, which then increased to 18.62% (at 3.53 eV) and 15.24% (at 4.30 eV) for CsPbBr_3_(x = 0.00) and CsPbCl_3_ (x = 1.00), respectively, as shown in Figure 16. The maximum reflectivity peaks of 48%, 46.7%, 47.8%, 48.5%, 48.7%, 48.6%, and 51% occurred at energy values of 15.88, 15.97, 16.00, 16.10, 16.16, 16.18, and 16.29 eV, respectively, and then began to fluctuate and decrease at higher energies. The value of R (0) decreased with the increase in Cl concentration (x), as shown in Figure 17 and presented in Table 3. The calculated R (0) versus Cl concentration (x) was fitted as follows:

(20) $$R\left(x\right)\%=\;10.3264-1.55348\;x-0.54961\;x^{2}.$$

**Figure 18:**
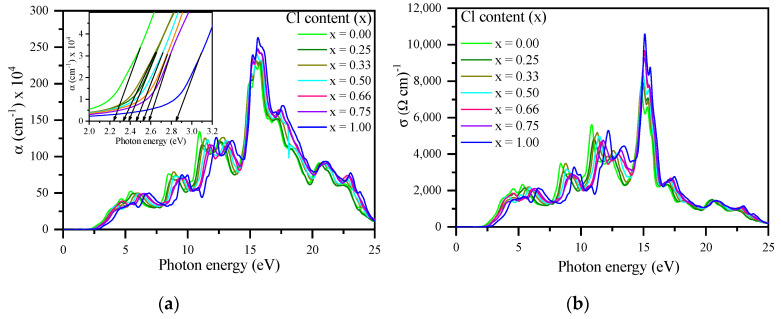
(**a**) Calculated absorption spectra *α*(*ω*) and (**b**) conductivity σ(ω) of CsPb(Br_1−x_Cl_x_)_3_ with respect to Cl content (x) using the mBJ–GGA potential. Inset: absorption spectra in the range from 2.0 to 3.2 eV.

Figure 18a shows the absorption coefficient α (ω). With the increase in Cl concentration (x), the absorption edge shifted to higher energy. The wide absorption range from visible to ultraviolet indicates that these compounds are useful for various optical and optoelectronic applications [72]. Figure 18b shows similar features of the optical conductivity *σ* (*ω*) characteristics, and provides information on the effects of external parameters on the electronic structure [118].

## 4. Conclusions

In this study, we investigated the influence of halide composition on the structural, electronic, and optical properties of the mixed-halide perovskites CsPb(Br_1−x_Cl_x_)_3_ using DFT. When the Cl content x was increased from 0.00 to 1.00, a decrease in unit-cell volume was observed. Theoretical XRD analyses revealed that the peak shifts to larger angles when the concentration of Cl increases. An increase in E_g_ was observed with an increase in the concentration of Cl. The E_g_ values calculated using the PBE–GGA potential were between 1.53 and 1.93 eV, while those calculated using the mBJ–GGA potential were between 2.23 and 2.90 eV. The increase in E_g_ with the increase in Cl content was due to the fact that the hybridization of Cl 3p states with Pb-s states was stronger than that with Br 4p states, which leads to a downshift of VBM and a decrease in the lattice constant. The calculated E_g_ and exciton binding energy E_b_ using mBJ–GGA potential best matched the previously reported experimental and theoretical values. The effective masses of electron and hole (m_e_* and m_h_*) are correlated with the energies of E_g_. The calculated photoabsorption coefficients display a blue shift of the absorption at a higher Cl concentration.

## Figures and Tables

**Table 1:** Calculated structural parameters; lattice constants a, b, and c (Å); unit cell volume V (Å)^3^; bulk modulus B (GPa); and its derivative B′ of CsPb(Br_1−x_Cl_x_)_3_ perovskite by Wu and Cohen generalized gradient approximation (WC–GGA) potential. mBJ, modified Becke−Johnson; LDA, local density approximation; PBE, Perdew–Burke–Ernzerhof.

CsPb(Br_1−x_Cl_x_)_3_	Present Work				Other Calculations (Exp.)		
	a (Å)	V (Å)^3^	B (GPa)	B′	a (Å)	B (GPa)	B′
**CsPbBr_3_**	a = 5.874	810.703	20.7379	4.881	5.84 (WC–GGA) [72] 5.86 (TB–mBJ) * [73] 5.74 (LDA) [74,75] 6.005 (PBE–GGA) [23] 5.87 (PBEsol) [23] 5.87 (PBE–GGA) [76] 5.77 (LDA) [23] 6.0039 (PBE–GGA) [77] (5.874) [36] (5.85) [69]	23.5 [72]	5.0 [72]
**CsPbBr_2.75_Cl_0.25_**	a = 5.801 c = 5.855	807.008			a = 6.005 c = 5.859 (PBE–GGA) [78]		
**CsPbBr_2_Cl**	a = 5.784 c = 5.748	774.139			a = 5.708 c = 6.012 (PBE–GGA) [78]		
**CsPbBr_1.5_Cl_1.5_**	a = 5.739 c = 5.7395	756.278			a = 5.718 c = 5.874 (PBE–GGA) [78]	-	-
**CsPbBrCl_2_**	a = 5.695 c = 5.6947	738.692			a = 5.725 c = 6.012 (PBE–GGA) [78]		
**CsPbBr_0.25_Cl_2.75_**	a = 5.672 c = 5.6722	730.005			a = 5.728 c = 5.879 (PBE–GGA) [78]		
**CsPbCl_3_**	a = 5.605	704.347	24.2106	5.0142	5.56 (WC–GGA) [72] 5.61 (TB–mBJ) [73] 5.73 (PBE–GGA) [79] 5.49 (LDA) [75] 5.743 (PBE–GGA) [80] 5.726 (PBE–GGA) [78] 5.728 (PBE–GGA) [81] 5.618 (PBE–GGA)[82] 5.740 (LDA) [74] 5.603 (PBE–GGA) [55] 5.605 [70,83] 5.61 [55] 5.6228 [30]	25.8 [72] 22.59 [81] 25.447[82] 26.33 [73]	5.0 [72] 4.33 [81] 4.4 [82]

* Tran and Blaha modified Becke-Johnson potential.

**Table 2:** Calculated E_g_ (eV) values of CsPb(Br_1−x_Cl_x_)_3_ perovskite using PBE–GGA, mBJ–GGA, and mBJ–GGA + spin-orbital coupling (SOC) potentials, and mBJ–GGA + SOC(C).

CsPb(Br_1−x_Cl_x_)_3_	E_g_ (eV)				
	This Work				Other (Exp.)
	PBE–GGA	mBJ–GGA	mBJ–GGA + SOC	mBJ–GGA + SOC (C)	
**CsPbBr_3_**	1.53	2.23	1.05	2.28	2.34 (GW) [74] 1.61 (PBE–GGA) [23] 2.36 (nTmBj) [23] 2.228 (KTB–mBJ) * [92] 2.08 (GLLB-SC) ** [93] 2.10 (QE) *** [35] 2.50 (mBJ–GGA) [77] (2.36) [51,94] (2.32) [52] (2.282) [53] (2.35) [95]
**CsPbBr_2.75_Cl_0.25_**	1.68	2.46	1.40	2.64	1.809 (PBE–GGA) [78]
**CsPbBr_2_Cl**	1.56	2.40	1.20	2.45	1.827 (PBE–GGA) [78] (2.59) [94]
**CsPbBr_1.5_Cl_1.5_**	1.69	2.51	1.41	2.67	1.859 (PBE–GGA) [78] (2.72) [94]
**CsPbBrCl_2_**	1.71	2.59	1.52	2.78	1.881(PBE–GGA) [78] (2.88) [94]
**CsPbBr_0.25_Cl_2.75_**	1.77	2.64	1.53	2.80	2.05(PBE–GGA) [78]
**CsPbCl_3_**	1.93	2.90	1.69	2.97	2.20 (PBE–GGA) [78,96] 2.829 (KTB–mBJ) [92] 2.92 (HSE) **** [79] 3.406 (PBE–GGA)[82] 2.88 (GW) [74] 2.74 (TB–mBJ) [73] 2.168 (PBE–GGA) [23] 3.10 (nTmBj) [23] (3.00) [54] (2.97) [55] (3.04) [95](2.98) [94]

* Koller, Tran, and Blaha modified Becke-Johnson potential; ** Gritsenko, van Leeuwen, van Lenthe, and Baerends-Solid and Correlation; *** Quantum Espresso 6.0; **** Hybrid nonlocal exchange-correlation functional.

**Table 3:** Calculation of static optical parameters ε_1_(0), refractive index n(0), and reflectivity R(0) for CsPb(Br_1−x_Cl_x_)_3_ compounds.

CsPb(Br_1−x_Cl_x_)_3_	mBJ–GGA (others)		
	ε_1_ (0)	n (0)	R (0)%
**CsPbBr_3_**	3.82 (4.30) [104] (4.60) [23] (4.63) [72]	1.96 Exp. (1.85–2.3) [56] (2.152) [72]	10.50 (13.4) [72]
**CsPbBr_2.75_Cl_0.25_**	3.59	1.897	9.65
**CsPbBr_2_Cl**	3.57	1.890	9.55
**CsPbBr_1.5_Cl_1.5_**	3.56	1.882	9.52
**CsPbBrCl_2_**	3.55	1.880	9.36
**CsPbBr_0.25_Cl_2.75_**	3.41	1.848	8.85
**CsPbCl_3_**	3.23 (3.69) [104] (3.00) [81] (4.10) [23] (4.43) [72]	1.798 (1.739) [81] (2.105) [72]	8.11 (12.7) [72] (10) [82]

## Supplementary Materials

The following are available online at https://www.mdpi.com/1996-1944/13/21/4944/s1, Tables S1–S7: Structural properties of CsPb(Br_1−x_Cl_x_)_3_ Perovskite, Table S8: Effective mass of electron (m_e_*) and hole (m_h_*), reduced mass (µ_r_), bohr diameter (a_0_), dielectric constant (ε), and exciton binding energy (E_b_) values calculated by PBE–GGA, mBJ–GGA, and mBJ–GGA + SOC potentials, Figure S1: Band structures and PDOS of (a) CsPbBr_3_ and (b) CsPbCl_3_ obtained using the mBJ–GGA potential.
