# Supplementary material for: Structural, Electronic, and Optical Properties of CsPb(Br1−xClx)3 Perovskite: First-Principles Study with PBE–GGA and mBJ–GGA Methods
Source: Materials (Basel). 2020 Nov 3;13(21):4944. doi: 10.3390/ma13214944 (PMC7662594; doi:10.3390/ma13214944)
Supplement: Supplementary file 1 [file materials-13-04944-s001.pdf]

materials

Supplementary Materials: Structural, Electronic, and Optical Properties of $CsPb(Br_{1-x}Cl_x)_3$ Perovskite: First-Principles Study with PBE–GGA and mBJ–GGA Methods

Hamid M. Ghaithan [1,*], Zeyad. A. Alahmed [1,*], Saif M. H. Qaid [1], and Abdullah S. Aldwayyan [1,2,3,*]

[1] Physics and Astronomy Department, College of Science, King Saud University, P.O. Box 2455, Riyadh 11451, Saudi Arabia; sqaid@ksu.edu.sa
[2] King Abdullah Institute for Nanotechnology, King Saud University, P.O. Box 2454, Riyadh 11451, Saudi Arabia
[3] K.A.CARE Energy Research and Innovation Center at Riyadh, P.O. Box 2022, Riyadh 11454, Saudi Arabia
* Correspondence: hghaithan@ksu.edu.sa (H.M.G.); zalahmed@ksu.edu.sa (Z.A.A.); dwayyan@ksu.edu.sa (A.S.A.); Tel.: +96-6532-257-491 (H.M.G.)

Table S1. CsPbBr3.

Lattice Parameters					
a	b	c	alpha	beta	gamma
5.87400	5.87400	23.49600	90.0000	90.0000	90.0000
Unit-cell volume = 810.703101 Å^3					

Structure Parameters								
		x	y	z	Occ.	B	Site	Sym.
1	Cs Cs1	0.00000	0.00000	0.00000	1.000	1.000	1a	1
2	Cs Cs2	0.00000	0.00000	0.25000	1.000	1.000	1a	1
3	Cs Cs3	0.00000	0.00000	0.50000	1.000	1.000	1a	1
4	Cs Cs4	0.00000	0.00000	0.75000	1.000	1.000	1a	1
5	Pb Pb1	0.50000	0.50000	0.12500	1.000	1.000	1a	1
6	Pb Pb2	0.50000	0.50000	0.37500	1.000	1.000	1a	1
7	Pb Pb3	0.50000	0.50000	0.62500	1.000	1.000	1a	1
8	Pb Pb4	0.50000	0.50000	0.87500	1.000	1.000	1a	1
9	Br Br1	0.50000	0.50000	1.00000	1.000	1.000	1a	1
10	Br Br2	0.00000	0.50000	0.12500	1.000	1.000	1a	1
11	Br Br3	0.50000	0.00000	0.12500	1.000	1.000	1a	1
12	Br Br4	0.50000	0.50000	0.25000	1.000	1.000	1a	1
13	Br Br5	0.00000	0.50000	0.37500	1.000	1.000	1a	1
14	Br Br6	0.50000	0.00000	0.37500	1.000	1.000	1a	1
15	Br Br7	0.50000	0.50000	0.50000	1.000	1.000	1a	1
16	Br Br8	0.00000	0.50000	0.62500	1.000	1.000	1a	1
17	Br Br9	0.50000	0.00000	0.62500	1.000	1.000	1a	1
18	Br Br10	0.50000	0.50000	0.75000	1.000	1.000	1a	1
19	Br Br11	0.00000	0.50000	0.87500	1.000	1.000	1a	1
20	Br Br12	0.50000	0.0000	0.87500	1.000	1.000	1a	1

Lattice type: P; Space group name: P 1; Space group number: 1; Setting number:1.

Table S2. $CsPbBr_{2.75}Cl_{0.25}$.

Lattice Parameters								
**a**	**b**	**c**	**alpha**	**beta**	**gamma**			
5. 80100	5.80100	23.42026	90.0000	90.0000	90.0000			
Unit-cell volume = 807.008322 Å^3								
**Structure Parameters**								
		**x**	**y**	**z**	**Occ.**	**B**	**Site**	**Sym.**
1	Cs Cs1	0.00000	0.00000	0.00000	1.000	1.000	1a	1
2	Cs Cs2	0.00000	0.00000	0.25000	1.000	1.000	1a	1
3	Cs Cs3	0.00000	0.00000	0.50000	1.000	1.000	1a	1
4	Cs Cs4	0.00000	0.00000	0.75000	1.000	1.000	1a	1
5	Pb Pb1	0.50000	0.50000	0.12144	1.000	1.000	1a	1
6	Pb Pb2	0.50000	0.50000	0.37437	1.000	1.000	1a	1
7	Pb Pb3	0.50000	0.50000	0.62563	1.000	1.000	1a	1
8	Pb Pb4	0.50000	0.50000	0.87856	1.000	1.000	1a	1
9	Cl Cl1	0.50000	0.50000	1.00000	1.000	1.000	1a	1
10	Br Br1	0.00000	0.50000	0.12488	1.000	1.000	1a	1
11	Br Br2	0.50000	0.00000	0.12486	1.000	1.000	1a	1
12	Br Br3	0.50000	0.50000	0.24829	1.000	1.000	1a	1
13	Cl Cl2	0.00000	0.50000	0.37504	1.000	1.000	1a	1
14	Br Br4	0.50000	0.00000	0.37507	1.000	1.000	1a	1
15	Br Br5	0.50000	0.50000	0.50000	1.000	1.000	1a	1
16	Br Br6	0.00000	0.50000	0.62493	1.000	1.000	1a	1
17	Cl Cl3	0.50000	0.00000	0.62496	1.000	1.000	1a	1
18	Br Br7	0.50000	0.50000	0.75171	1.000	1.000	1a	1
19	Br Br8	0.00000	0.50000	0.87514	1.000	1.000	1a	1
20	Br Br9	0.50000	0.00000	0.87512	1.000	1.000	1a	1

Lattice type: P; Space group name: P 1; Space group number: 1; Setting number:1.

Table S3. $\mathbf{CsPbBr_2Cl}$

Lattice Parameters								
**a**	**b**	**c**	**alpha**	**beta**	**gamma**			
5.78433	5.78433	23.13731	90.0000	90.0000	90.0000			
Unit-cell volume = 774.138727 Å^3								
Structure Parameters								
		x	y	z	Occ	B	Site	Sym.
1	Cs Cs1	0.00000	0.00000	0.00000	1.000	1.000	1a	1
2	Cs Cs2	0.00000	0.00000	0.25000	1.000	1.000	1a	1
3	Cs Cs3	0.00000	0.00000	0.50000	1.000	1.000	1a	1
4	Cs Cs4	0.00000	0.00000	0.75000	1.000	1.000	1a	1
5	Pb Pb1	0.50000	0.50000	0.12500	1.000	1.000	1a	1
6	Pb Pb2	0.50000	0.50000	0.37500	1.000	1.000	1a	1
7	Pb Pb3	0.50000	0.50000	0.62500	1.000	1.000	1a	1
8	Pb Pb4	0.50000	0.50000	0.87500	1.000	1.000	1a	1
9	Br Br1	0.50000	0.50000	0.00000	1.000	1.000	1a	1
10	Br Br2	0.00000	0.50000	0.12500	1.000	1.000	1a	1
11	Cl Cl1	0.50000	0.00000	0.12500	1.000	1.000	1a	1
12	Br Br3	0.50000	0.50000	0.25000	1.000	1.000	1a	1
13	Br Br4	0.00000	0.50000	0.37500	1.000	1.000	1a	1
14	Cl Cl2	0.50000	0.00000	0.37500	1.000	1.000	1a	1
15	Br Br5	0.50000	0.50000	0.50000	1.000	1.000	1a	1

16	Br Br6	0.00000	0.50000	0.62500	1.000	1.000	1a	1
17	Cl Cl3	0.50000	0.00000	0.62500	1.000	1.000	1a	1
18	Br Br7	0.50000	0.50000	0.75000	1.000	1.000	1a	1
19	Br Br8	0.00000	0.50000	0.87500	1.000	1.000	1a	1
20	Cl Cl4	0.50000	0.00000	0.87500	1.000	1.000	1a	1

Lattice type: P; Space group name: P 1; Space group number: 1; Setting number:1.

Table S4. $CsPbBr_{1.5}Cl_{1.5}$

Lattice Parameters					
a	b	c	alpha	beta	gamma
5.73950	5.73950	22.95799	90.0000	90.0000	90.0000
Unit-cell volume = 756.278366 Å^3					

Structure Parameters								
		x	y	z	Occ	B	Site	Sym.
1	Cs Cs1	0.00000	0.00000	0.00000	1.000	1.000	1a	1
2	Cs Cs2	0.00000	0.00000	0.25000	1.000	1.000	1a	1
3	Cs Cs3	0.00000	0.00000	0.50000	1.000	1.000	1a	1
4	Cs Cs4	0.00000	0.00000	0.75000	1.000	1.000	1a	1
5	Pb Pb1	0.50000	0.50000	0.12500	1.000	1.000	1a	1
6	Pb Pb2	0.50000	0.50000	0.37500	1.000	1.000	1a	1
7	Pb Pb3	0.50000	0.50000	0.62500	1.000	1.000	1a	1
8	Pb b4	0.50000	0.50000	0.87500	1.000	1.000	1a	1
9	Cl Cl1	0.50000	0.50000	1.00000	1.000	1.000	1a	1
10	Br Br1	0.00000	0.50000	0.12490	1.000	1.000	1a	1
11	Cl Cl2	0.50000	0.00000	0.12495	1.000	1.000	1a	1
12	Br Br2	0.50000	0.50000	0.25000	1.000	1.000	1a	1
13	Cl Cl3	0.00000	0.50000	0.37505	1.000	1.000	1a	1
14	Br Br3	0.50000	0.00000	0.37510	1.000	1.000	1a	1
15	Cl Cl4	0.50000	0.50000	0.50000	1.000	1.000	1a	1
16	Br Br4	0.00000	0.50000	0.62490	1.000	1.000	1a	1
17	Cl Cl5	0.50000	0.00000	0.62495	1.000	1.000	1a	1
18	Br Br5	0.50000	0.50000	0.75000	1.000	1.000	1a	1
19	Cl Cl6	0.00000	0.50000	0.87505	1.000	1.000	1a	1
20	Br Br6	0.50000	0.00000	0.87510	1.000	1.000	1a	1

Lattice type: P; Space group name: P 1; Space group number: 1; Setting number:1.

Table S5. $CsPbBrCl_2$.

Lattice Parameters					
a	b	c	alpha	beta	gamma
5.69466	5.69466	22.77865	90.0000	90.0000	90.0000
Unit-cell volume = 738.691816 Å^3					

Structure Parameters								
		x	y	z	Occ.	B	Site	Sym.
1	Cs Cs1	0.00000	0.00000	0.00000	1.000	1.000	1a	1
2	Cs Cs2	0.00000	0.00000	0.25000	1.000	1.000	1a	1
3	Cs Cs3	0.00000	0.00000	0.50000	1.000	1.000	1a	1
4	Cs Cs4	0.00000	0.00000	0.75000	1.000	1.000	1a	1
5	Pb Pb1	0.50000	0.50000	0.12500	1.000	1.000	1a	1

6	Pb Pb2	0.50000	0.50000	0.37500	1.000	1.000	1a	1
7	Pb Pb3	0.50000	0.50000	0.62500	1.000	1.000	1a	1
8	Pb Pb4	0.50000	0.50000	0.87500	1.000	1.000	1a	1
9	Cl Cl1	0.50000	0.50000	1.00000	1.000	1.000	1a	1
10	Cl Cl2	0.00000	0.50000	0.12500	1.000	1.000	1a	1
11	Br Br1	0.50000	0.00000	0.12500	1.000	1.000	1a	1
12	Cl Cl3	0.50000	0.50000	0.25000	1.000	1.000	1a	1
13	Cl Cl4	0.00000	0.50000	0.37500	1.000	1.000	1a	1
14	Br Br2	0.50000	0.00000	0.37500	1.000	1.000	1a	1
15	Cl Cl5	0.50000	0.50000	0.50000	1.000	1.000	1a	1
16	Cl Cl6	0.00000	0.50000	0.62500	1.000	1.000	1a	1
17	Br Br3	0.50000	0.00000	0.62500	1.000	1.000	1a	1
18	Cl Cl7	0.50000	0.50000	0.75000	1.000	1.000	1a	1
19	Cl Cl8	0.00000	0.50000	0.87500	1.000	1.000	1a	1
20	Br Br4	0.50000	0.00000	0.87500	1.000	1.000	1a	1

Lattice type: P; Space group name: P 1; Space group number: 1; Setting number:1.

Table S6. $CsPbBr_{0.25}Cl_{2.75}$.

Lattice Parameters								
a	b	c	alpha	beta	gamma			
5.67225	5.67225	22.68899	90.0000	90.0000	90.0000			
Unit-cell volume = 730.004601 Å^3								
**Structure Parameters**								
		**x**	**y**	**z**	**Occ.**	**B**	**Site**	**Sym.**
1	Cs Cs1	0.00000	0.00000	0.00000	1.000	1.000	1a	1
2	Cs Cs2	0.00000	0.00000	0.25000	1.000	1.000	1a	1
3	Cs Cs3	0.00000	0.00000	0.50000	1.000	1.000	1a	1
4	Cs Cs4	0.00000	0.00000	0.75000	1.000	1.000	1a	1
5	Pb Pb1	0.50000	0.50000	0.12848	1.000	1.000	1a	1
6	Pb Pb2	0.50000	0.50000	0.37562	1.000	1.000	1a	1
7	Pb Pb3	0.50000	0.50000	0.62438	1.000	1.000	1a	1
8	Pb Pb4	0.50000	0.50000	0.87153	1.000	1.000	1a	1
9	Br Br1	0.50000	0.50000	1.00000	1.000	1.000	1a	1
10	Cl Cl1	0.00000	0.50000	0.12521	1.000	1.000	1a	1
11	Cl Cl2	0.50000	0.00000	0.12523	1.000	1.000	1a	1
12	Cl Cl3	0.50000	0.50000	0.25166	1.000	1.000	1a	1
13	Br Br2	0.00000	0.50000	0.37492	1.000	1.000	1a	1
14	Cl Cl4	0.50000	0.00000	0.37493	1.000	1.000	1a	1
15	Cl Cl5	0.50000	0.50000	0.50000	1.000	1.000	1a	1
16	Cl Cl6	0.00000	0.50000	0.62507	1.000	1.000	1a	1
17	Br Br3	0.50000	0.00000	0.62508	1.000	1.000	1a	1
18	Cl Cl7	0.50000	0.50000	0.74834	1.000	1.000	1a	1
19	Cl Cl8	0.00000	0.50000	0.87477	1.000	1.000	1a	1
20	Cl Cl9	0.50000	0.00000	0.87479	1.000	1.000	1a	1

Lattice type: P; Space group name: P 1; Space group number: 1; Setting number:1.

Table S7. $CsPbCl_3$.

Lattice Parameters					
a	b	c	alpha	beta	gamma
5.60500	5.60500	22.41999	90.0000	90.0000	90.0000
Unit-cell volume = 704.346569 Å^3					

Structure Parameters								
		**x**	**y**	**z**	**Occ.**	**B**	**Site**	**Sym.**
1	Cs Cs1	0.00000	0.00000	0.00000	1.000	1.000	1a	1
2	Cs Cs2	0.00000	0.00000	0.25000	1.000	1.000	1a	1
3	Cs Cs3	0.00000	0.00000	0.50000	1.000	1.000	1a	1
4	Cs Cs4	0.00000	0.00000	0.75000	1.000	1.000	1a	1
5	Pb Pb1	0.50000	0.50000	0.12500	1.000	1.000	1a	1
6	Pb Pb2	0.50000	0.50000	0.37500	1.000	1.000	1a	1
7	Pb Pb3	0.50000	0.50000	0.62500	1.000	1.000	1a	1
8	Pb Pb4	0.50000	0.50000	0.87500	1.000	1.000	1a	1
9	Cl Cl1	0.50000	0.50000	1.00000	1.000	1.000	1a	1
10	Cl Cl2	0.00000	0.50000	0.12500	1.000	1.000	1a	1
11	Cl Cl3	0.50000	0.00000	0.12500	1.000	1.000	1a	1
12	Cl Cl4	0.50000	0.50000	0.25000	1.000	1.000	1a	1
13	Cl Cl5	0.00000	0.50000	0.37500	1.000	1.000	1a	1
14	Cl Cl6	0.50000	0.00000	0.37500	1.000	1.000	1a	1
15	Cl Cl7	0.50000	0.50000	0.50000	1.000	1.000	1a	1
16	Cl Cl8	0.00000	0.50000	0.62500	1.000	1.000	1a	1
17	Cl Cl9	0.50000	0.00000	0.62500	1.000	1.000	1a	1
18	Cl Cl10	0.50000	0.50000	0.75000	1.000	1.000	1a	1
19	Cl Cl11	0.00000	0.50000	0.87500	1.000	1.000	1a	1
20	Cl Cl12	0.50000	0.00000	0.87500	1.000	1.000	1a	1

Lattice type: P; Space group name: P 1; Space group number: 1; Setting number:1.

Table S8. Effective mass of electron (m_e^*) and hole (m_h^*), reduced mass (μ_r), bohr diameter (a_0), dielectric constant (ε), and exciton binding energy (E_b) values calculated by PBE–GGA, mBJ– GGA, and mBJ-GGA + SOC potentials.

**Perovskites**	m_e^*	m_h^*	μ_r	**a_0 (nm)**	**ε(0)**	**E_b (meV)**
**$CsPbBr_3$**	$0.215^{\text{PBE–GGA}}$<br>$0.283^{\text{mBJ–GGA}}$<br>$0.049^{\text{mBJ + SOC}}$<br>0.22 [1]<br>0.2 [2]<br>0.15 [3]<br>0.149 [4]<br>0.26 [5]<br>0.15 [6]	$0.043^{\text{PBE–GGA}}$<br>$0.053^{\text{mBJ–GGA}}$<br>$0.045^{\text{mBJ + SOC}}$<br>0.14 [1,3]<br>0.12 [2,5]<br>0.143 [4]<br>0.23 [5]<br>0.10 [5]<br>0.14 [6]	$0.036^{\text{PBE–GGA}}$<br>$0.045^{\text{mBJ–GGA}}$<br>$0.023^{\text{mBJ + SOC}}$	$12.5^{\text{PBE–GGA}}$<br>$8.1^{\text{mBJ–GGA}}$<br>5.5 [5]<br>4.2 [5]<br>7 [3]<br>$5.8^{\text{our paper}}$	$4.69^{\text{PBE–GGA}}$<br>$3.82^{\text{mBJ–GGA}}$<br>4.631 [7]<br>4.96 [3]	$22^{\text{PBE–GGA}}$<br>$42^{\text{mBJ–GGA}}$<br>$58^{\text{our paper}}$<br>70 [5]<br>106 [5]<br>40 [3]<br>35 [8]<br>40 [9]
**$CsPbBr_{2.75}Cl_{0.25}$**	$0.133^{\text{PBE–GGA}}$<br>$0.125^{\text{mbj–GGA}}$<br>$0.061^{\text{mBJ + SOC}}$	$0.047^{\text{PBE–GGA}}$<br>$0.058^{\text{mBJ–GGA}}$<br>$0.054^{\text{mBJ + SOC}}$	$0.035^{\text{PBE–GGA}}$<br>$0.039^{\text{mBJ–GGA}}$<br>$0.029^{\text{mBJ + SOC}}$	$11.8^{\text{PBE–GGA}}$<br>$8.8^{\text{mBJ–GGA}}$	$4.34^{\text{PBE–GGA}}$<br>$3.59^{\text{mBJ–GGA}}$	$25^{\text{PBE–GGA}}$<br>$41^{\text{mBJ–GGA}}$
**$CsPbBr_2Cl$**	$0.119^{\text{PBE–GGA}}$<br>$0.346^{\text{mBJ–GGA}}$<br>$0.067^{\text{mBJ + SOC}}$	$0.058^{\text{PBE–GGA}}$<br>$0.073^{\text{mBJ–GGA}}$<br>$0.063^{\text{mBJ + SOC}}$	$0.027^{\text{PBE–GGA}}$<br>$0.060^{\text{mBJ–GGA}}$<br>$0.032^{\text{mBJ + SOC}}$	$14.9^{\text{PBE–GGA}}$<br>$5.7^{\text{mBJ–GGA}}$	$4.23^{\text{PBE–GGA}}$<br>$3.57^{\text{mBJ–GGA}}$	$21^{\text{PBE–GGA}}$<br>$46^{\text{mBJ–GGA}}$
**$CsPbBr_{1.5}Cl_{1.5}$**	$0.197^{\text{PBE–GGA}}$<br>$0.255^{\text{mBJ–GGA}}$<br>$0.064^{\text{mBJ + SOC}}$	$0.046^{\text{PBE–GGA}}$<br>$0.057^{\text{mBJ–GGA}}$<br>$0.055^{\text{mBJ + SOC}}$	$0.037^{\text{PBE–GGA}}$<br>$0.047^{\text{mBJ–GGA}}$<br>$0.029^{\text{mBJ + SOC}}$	$10.6^{\text{PBE–GGA}}$<br>$7.2^{\text{mBJ–GGA}}$	$4.12^{\text{PBE–GGA}}$<br>$3.56^{\text{mBJ–GGA}}$	$30^{\text{PBE–GGA}}$<br>$50^{\text{mBJ–GGA}}$
**$CsPbBrCl_2$**	$0.202^{\text{PBE–GGA}}$<br>$0.255^{\text{mBJ–GGA}}$<br>$0.054^{\text{mBJ + SOC}}$	$0.037^{\text{PBE–GGA}}$<br>$0.045^{\text{mBJ–GGA}}$<br>$0.045^{\text{mBJ + SOC}}$	$0.031^{\text{PBE–GGA}}$<br>$0.038^{\text{mBJ–GGA}}$<br>$0.025^{\text{mBJ + SOC}}$	$12.6^{\text{PBE–GGA}}$<br>$8.9^{\text{mBJ–GGA}}$	$4.10^{\text{PBE–GGA}}$<br>$3.55^{\text{mBJ–GGA}}$	$25^{\text{PBE–GGA}}$<br>$41^{\text{mBJ–GGA}}$
**$CsPbBr_{0.25}Cl_{2.75}$**	$0.197^{\text{PBE–GGA}}$<br>$0.178^{\text{mBJ–GGA}}$<br>$0.067^{\text{mBJ + SOC}}$	$0.046^{\text{PBE–GGA}}$<br>$0.057^{\text{mBJ–GGA}}$<br>$0.054^{\text{mBJ + SOC}}$	$0.037^{\text{PBE–GGA}}$<br>$0.043^{\text{mBJ–GGA}}$<br>$0.030^{\text{mBJ + SOC}}$	$10.0^{\text{PBE–GGA}}$<br>$7.6^{\text{mBJ–GGA}}$	$3.89^{\text{PBE–GGA}}$<br>$3.4^{\text{mBJ–GGA}}$	$33^{\text{PBE–GGA}}$<br>$50^{\text{mBJ– GGA}}$

**$CsPbCl_3$**	$0.237^{PBE–GGA}$<br>$0.312^{mBJ–GGA}$<br>$0.073^{mBJ + SOC}$<br>0.28 [1]<br>0.218 [4]<br>0.23 [5]<br>0.20 [3]	$0.053^{PBE–GGA}$<br>$0.067^{mBJ–GGA}$<br>$0.061^{mBJ + SOC}$<br>0.18 [1]<br>0.189 [4]<br>0.10 [5]<br>0.17 [3]	$0.043^{PBE–GGA}$<br>$0.055^{mBJ–GGA}$<br>$0.033^{mBJ + SOC}$	$8.6^{PBE–GGA}$<br>$5.6^{mBJ–GGA}$<br>4.1 [5]<br>5.5 [5]<br>5 [3]	$3.87^{PBE–GGA}$<br>$3.23^{mBJ–GGA}$<br>4.1 [7]<br>2.723 [5]<br>3.09 [5]<br>4.07 [3]	$39^{PBE–GGA}$<br>$72^{mBJ–GGA}$<br>130 [5]<br>85 [5]<br>75 [3]

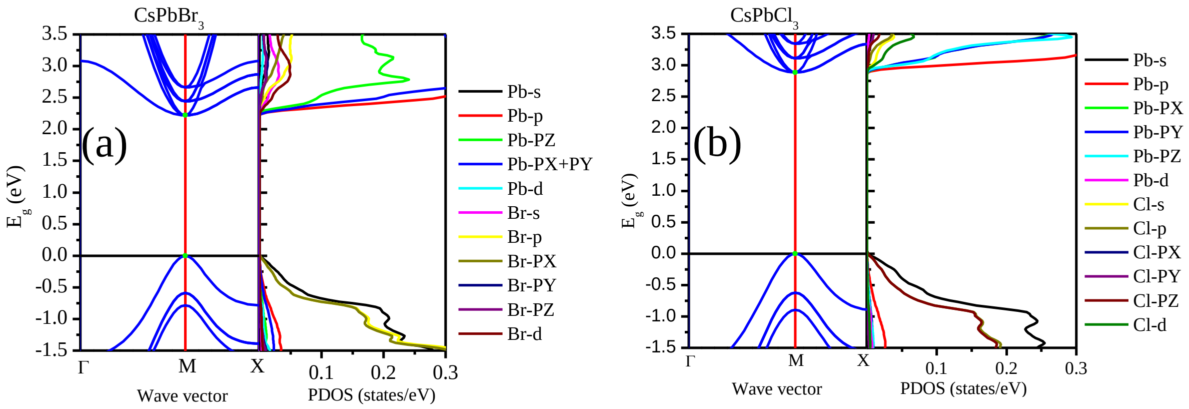

Figure S1. Band structures and PDOS of (**a**) $CsPbBr_3$ and (**b**) $CsPbCl_3$ obtained using the mBJ–GGA potential.

References

1. Qian, J.; Xu, B.; Tian, W. A comprehensive theoretical study of halide perovskites ABX3. *Org. Electron.* **2016**, *37*, 61–73, doi:10.1016/j.orgel.2016.05.046.
2. Chang, Y.H.; Park, C.H. First-Principles Study of the Structural and the Electronic Properties of the CsPbX3 and CH3NH3PbX3. *J. Korean Phys. Soc*. **2004**, *44*, 889–893.
3. Protesescu, L.; Yakunin, S.; Bodnarchuk, M.I.; Krieg, F.; Caputo, R.; Hendon, C.H.; Yang, R.X.; Walsh, A.; Kovalenko, M.V. Nanocrystals of Cesium Lead Halide Perovskites (CsPbX3, X = Cl, Br, and I): Novel Optoelectronic Materials Showing Bright Emission with Wide Color Gamut. *Nano Lett.* **2015**, *15*, 3692–3696, doi:10.1021/nl5048779.
4. Kang, Y.; Han, S. Intrinsic Carrier Mobility of Cesium Lead Halide Perovskites. *Phys. Rev. Appl.* **2018**, *10*, 044013, doi:10.1103/physrevapplied.10.044013.
5. Tomanová, K.; Čuba, V.; Brik, M.G.; Mihóková, E.; Turtos, R.M.; Lecoq, P.; Auffray, E.; Nikl, M. On the structure, synthesis, and characterization of ultrafast blue-emitting CsPbBr3 nanoplatelets. *APL Mater.* **2019**, *7*, 011104, doi:10.1063/1.5079300.
6. Wu, K.; Liang, G.; Shang, Q.; Ren, Y.; Kong, D.; Lian, T. Ultrafast Interfacial Electron and Hole Transfer from CsPbBr3 Perovskite Quantum Dots. *J. Am. Chem. Soc.* **2015**, *137*, 12792–12795, doi:10.1021/jacs.5b08520.
7. Ahmad, M.; Rehman, G.; Ali, L.; Shafiq, M.; Iqbal, R.; Ahmad, R.; Khan, T.; Jalali-Asadabadi, S.; Maqbool, M.; Ahmad, I. Structural, electronic and optical properties of CsPbX3 (X=Cl, Br, I) for energy storage and hybrid solar cell applications. *J. Alloy. Compd.* **2017**, *705*, 828–839, doi:10.1016/j.jallcom.2017.02.147.
8. Li, J.; Yuan, X.; Jing, P.; Li, J.; Wei, M.; Hua, J.; Zhao, J.; Tian, L. Temperature-dependent photoluminescence of inorganic perovskite nanocrystal films. *RSC Adv.* **2016**, *6*, 78311–78316, doi:10.1039/c6ra17008k.
9. Du, W.; Zhang, S.; Shi, J.; Chen, J.; Wu, Z.; Mi, Y.; Liu, Z.; Li, Y.; Sui, X.; Wang, R.; et al. Strong Exciton–Photon Coupling and Lasing Behavior in All-Inorganic CsPbBr3 Micro/Nanowire Fabry-Pérot Cavity. *ACS Photon-* **2018**, *5*, 2051–2059, doi:10.1021/acsphotonics.7b01593.

Publisher's Note: MDPI stays neutral with regard to jurisdictional claims in published maps and institutional affiliations.

© 2020 by the authors. Submitted for possible open access publication under the terms and conditions of the Creative Commons Attribution (CC BY) license (http://creativecommons.org/licenses/by/4.0/).
